# Proteolytic cleavage of pertussis toxin S1 subunit is not essential for its activity in mammalian cells

**DOI:** 10.1186/1471-2180-5-7

**Published:** 2005-02-03

**Authors:** Nicholas H Carbonetti, R Michael Mays, Galina V Artamonova, Roger D Plaut, Zoë EV Worthington

**Affiliations:** 1 Department of Microbiology and Immunology, University of Maryland School of Medicine, Baltimore, MD 21201, USA

## Abstract

**Background:**

Pertussis toxin (PT) is an exotoxin virulence factor produced by *Bordetella pertussis*, the causative agent of whooping cough. PT consists of an active subunit (S1) that ADP-ribosylates the alpha subunit of several mammalian G proteins, and a B oligomer (S2–S5) that binds glycoconjugate receptors on cells. PT appears to enter cells by endocytosis, and retrograde transport through the Golgi apparatus may be important for its cytotoxicity. A previous study demonstrated that proteolytic processing of S1 occurs after PT enters mammalian cells. We sought to determine whether this proteolytic processing of S1 is necessary for PT cytotoxicity.

**Results:**

Protease inhibitor studies suggested that S1 processing may involve a metalloprotease, and processing does not involve furin, a mammalian cell protease that cleaves several other bacterial toxins. However, inhibitor studies showed a general lack of correlation of S1 processing with PT cellular activity. A combination of replacement, insertion and deletion mutations in the C-terminal region of S1, as well as mass spectrometry data, suggested that the cleavage site is located around residue 203–204, but that cleavage is not strongly sequence-dependent. Processing of S1 was abolished by each of 3 overlapping 8 residue deletions just downstream of the putative cleavage site, but not by smaller deletions in the same region. Processing of the various mutant forms of PT did not correlate with cellular activity of the toxin, nor with the ability of the bacteria producing them to infect the mouse respiratory tract. In addition, S1 processing was not detected in transfected cells expressing S1, even though S1 was fully active in these cells.

**Conclusions:**

S1 processing is not essential for the cellular activity of PT. This distinguishes it from the processing of various other bacterial toxins, which has been shown to be important for their cytotoxicity. S1 processing may be mediated primarily by a metalloprotease, but the cleavage site on S1 is not sequence-dependent and processing appears to depend on the general topology of the protein in that region, indicating that multiple proteases may contribute to this cleavage.

## Background

Pertussis toxin (PT) is a complex exotoxin and an important virulence factor produced by *Bordetella pertussis*, a bacterial pathogen of the human respiratory tract that causes the disease whooping cough. PT holotoxin is a multi-subunit complex with an AB_5 _structure [1,2]: the enzymatically active A subunit (S1) is an ADP-ribosyltransferase that modifies the alpha subunit of several heterotrimeric G proteins (primarily G_i _proteins) in mammalian cells [3,4], and the B oligomer (S2, S3, 2 copies of S4, and S5) binds unidentified glycoconjugate receptors on cells [5,6]. The events in the intracellular trafficking of PT between surface binding and ADP-ribosylation of target G proteins on the cytoplasmic side of cellular membranes are relatively obscure. Electron microscopy studies and experiments with inhibitors suggest that the holotoxin is internalized by endocytosis [7-9]. Subcellular fractionation experiments and inhibition of cytotoxicity by Brefeldin A (BFA), which disrupts the Golgi apparatus [10], provide evidence for subsequent retrograde transport of PT to the Golgi apparatus [7-9]. Trafficking of PT beyond the Golgi apparatus is relatively uncharacterized, though it has been hypothesized that further retrograde transport of PT through the secretory pathway to the endoplasmic reticulum (ER) occurs [11-13]. After dissociation of S1 from the holotoxin, the liberated S1 subunit is then proposed to traverse the ER membrane to gain access to its target G proteins in the cytosol [13]. Evidence supporting this ER-to-cytosol translocation was obtained from transfection studies with constructs expressing S1 with a signal peptide for ER localization [12].

Another observation that may bear on the cell biology and cytotoxicity of PT is that the S1 subunit appears to be proteolytically processed to a lower molecular weight form upon interaction of PT with mammalian cells [14]. This processing was shown to be dependent upon entry of PT into cells and seemed to involve an early endosome function. The size of the processed form of S1 (approximately 22 kDa versus 26 kDa for the full-length S1) suggested that processing may be targeted at a protease-sensitive loop near the C-terminus of S1 that contains primary sites for trypsin and chymotrypsin cleavage [15]. However, evidence for the location of the cellular cleavage site on S1 was not presented. In addition, a link between processing of S1 and activity of PT in cells was not established. Proteolytic processing is a common theme in the activation of bacterial toxins upon interaction with mammalian cells. For example, anthrax toxin, diphtheria toxin, *Pseudomonas *exotoxin A and shiga toxin are all activated after cleavage by the endogenous eukaryotic protease furin [16], a subtilisin-like protease residing in the secretory pathway of eukaryotic cells [17], or by closely-related proteases [18]. Cholera toxin (CT) and *Escherichia coli *heat-labile toxin (LT) A subunits are cleaved at a protease-sensitive loop to promote maximal activity [19,20], and CT A subunit was found to be cleaved upon interaction of CT with T84 epithelial cells, by an unidentified protease [21].

In this study we extend the analysis of proteolytic processing of cell-associated S1 and conclude that S1 processing is not essential for the cellular activity of PT.

## Results and discussion

### Processing and fractionation of S1 in PT-treated CHO cells

**Figure 1:**
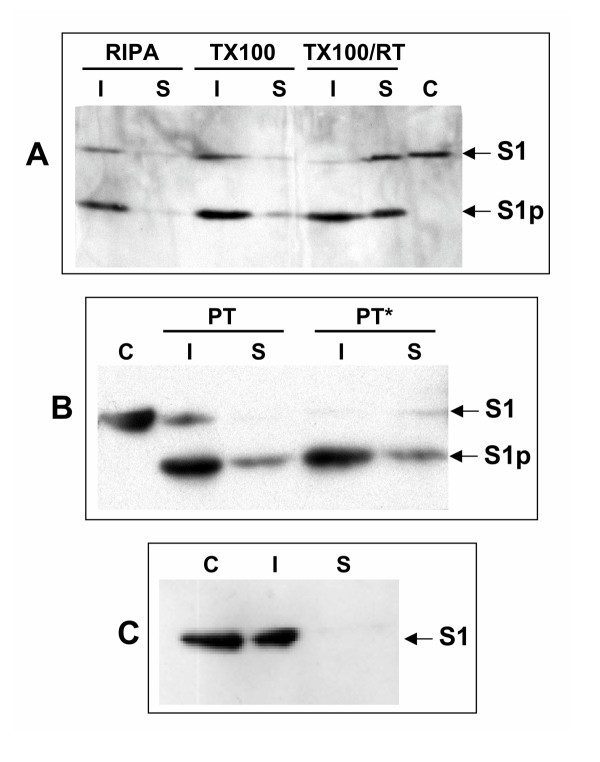
S1 processing and detergent fractionation in CHO cells. (A) Cells were incubated with PT (20 nM) for 4 h followed by lysis with RIPA or Triton X-100 (TX100) buffer on ice or at room temperature (RT). A western blot of the detergent-insoluble (I) and -soluble (S) fractions is shown, with bands corresponding to full length S1 or processed S1 (S1p) indicated. Lane marked C is PT (200 ng) loading control. (B) Western blot of Triton X-100 lysate fractions of CHO cells treated with PT or PT* (catalytically inactive mutant of PT) for 4 h. (C) Western blot of Triton X-100 lysate fractions of untreated CHO cells after addition of 500 ng PT to the lysis mixture.

In a previous study, ^125^I-labelled PT was used for analysis of S1 processing in mammalian cells [14]. As an alternative to radiolabeled toxin, we analyzed detergent lysates of cells treated with unlabeled PT to determine whether we could detect S1 processing. Near-confluent Chinese hamster ovary (CHO) cells were treated with PT (20 nM) for 4 h at 37°C, and then cells were washed, recovered by trypsinization and lysed on ice with either Triton X-100 lysis buffer or RIPA lysis buffer. The detergent-soluble and -insoluble fractions were then analyzed by sodium dodecylsulfate polyacrylamide gel electrophoresis (SDS-PAGE) and western blotting. As shown in Fig. 1A, we were able to detect processing of S1 by this method, with the majority of cell-associated S1 present as the lower molecular weight form (S1p, approximately 22 kDa, versus 26 kDa for full length S1). This processing event was independent of PT enzymatic activity, since the enzymatically inactive PT-9K/129G (PT*) was similarly processed by CHO cells (Fig. 1B). The kinetics of S1 processing (data not shown) were very similar to those previously reported [14], which, along with the similar size of the processed form, strongly suggested that we were observing the same event as previously studied. Surprisingly, however, the great majority (>80%) of S1 fractionated with the detergent-insoluble pellet material in this assay. Triton lysis at 25°C rather than on ice increased the proportion of S1 that was solubilized, but at least half of the processed form remained in the insoluble pellet material (Fig. 1A). To determine whether S1 fractionation with the detergent-insoluble material represented a potentially interesting feature of its intracellular transport, or merely an artifact of the lysis procedure, we added 500 ng PT to a Triton lysate of untreated CHO cells, incubated this on ice 30 min, centrifuged to separate the mix into soluble and insoluble fractions and analyzed these by SDS-PAGE and western blotting. Almost all (>95%) of the S1 fractionated to the insoluble pellet (Fig. 1C), demonstrating that S1 association with detergent-insoluble material occurs in the lysate and is independent of PT transport within cells. The Triton-insoluble fraction typically contains nuclei and cytoskeletal components [22], so it is possible that PT, or at least S1, has an affinity for one or more of these components.

### Effects of protease and cell trafficking inhibitors on S1 processing in CHO cells

**Figure 2:**
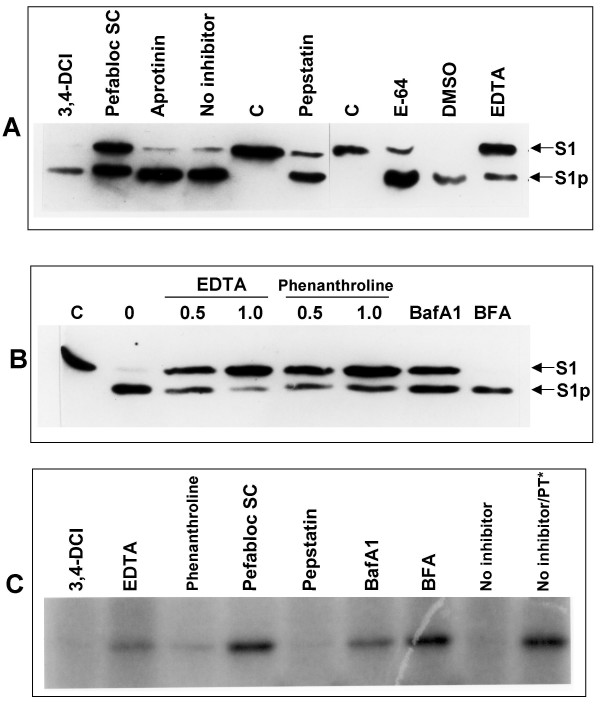
Effect of inhibitors on S1 processing and PT activity in CHO cells. (A, B) CHO cells were preincubated with the indicated inhibitor before addition of PT (20 nM) and then analyzed for S1 processing as before. Western blots are shown with unprocessed and processed (S1p) forms of S1 indicated. Lanes marked C are PT (100–200 ng) loading controls. DMSO = dimethylsulfoxide (solvent control). Metalloprotease inhibitor concentrations are indicated (mM). 0 = no inhibitor. BafA1 = bafilomycin A_1_. (C) Effect of inhibitors on ADP-ribosylation of CHO cell G proteins by PT. Autoradiogram of cellular ADP-ribosylation assay after treatment of CHO cells with the indicated inhibitor and either PT or PT* (last lane). Radiolabeled band (approximately 41 kDa) corresponds presumably with G_i_α2 and G_i_α3 [9].

We first ruled out the possibility that furin, a protease that cleaves several other bacterial toxins in mammalian cells, is responsible for S1 cleavage, by finding that S1 processing occurs normally in furin-deficient FD11 cells [23] (data not shown). This observation was not surprising since there are no consensus furin cleavage sites in the S1 sequence. In order to determine the catalytic type of protease responsible for S1 processing in CHO cells, we preincubated CHO cells with serine, cysteine, aspartic and metalloprotease inhibitors (following the suggestions of Barrett [24]) before addition of PT, and studied S1 processing by these cells as before (Fig. 2). The broad specificity serine protease inhibitor 3,4-DCI did not significantly inhibit processing (though the inhibitor was somewhat toxic to the CHO cells and reduced the amount of S1 recovered), and neither did the serine protease inhibitor aprotinin (Fig. 2A). The serine protease inhibitor pefabloc SC (BMB/Roche) did have a consistent inhibitory effect on S1 processing, with approximately 45% of the cell-associated S1 in the unprocessed form (versus approximately 10% on average in the absence of inhibitor). Since the other serine protease inhibitors had no effect on S1 processing, the reason for the inhibitory effect of pefabloc SC is unclear, but may be related to its ability to bind covalently to proteins (BMB/Roche). Neither the aspartic protease inhibitor pepstatin nor the cysteine protease inhibitor E-64 had any significant inhibitory effect on S1 processing (Fig. 2A) (nor did the cysteine/serine protease inhibitor leupeptin – data not shown). However, EDTA had a strong inhibitory effect on S1 processing by CHO cells (Fig. 2A,B), with 56% and 74% of cell-associated S1 in the unprocessed form in the presence of 0.5 mM and 1 mM EDTA, respectively. Since the inhibitory effect of a non-specific metal chelator such as EDTA could be due to the cation-dependence of activity of other proteases [24], we also used the metalloprotease inhibitor 1,10-phenanthroline, which has a high affinity for zinc and is considered the most useful inhibitor for metalloproteases [24]. This inhibitor also had a strong inhibitory effect on S1 processing by CHO cells (Fig. 2B), with 61% and 63% of cell-associated S1 in the unprocessed form in the presence of 0.5 mM and 1 mM phenanthroline, respectively. Therefore, we conclude that the cellular protease responsible for S1 processing in CHO cells is most likely a metalloprotease, which presumably resides in the secretory (endocytic) pathway and the identity of which remains to be determined. This is a novel observation in the sense that other bacterial toxins are cleaved by cellular proteases of the subtilisin family (such as furin) [16,23] or by other serine proteases [21], although one report demonstrated that CT activity on several different cell types was blocked by a competitive substrate for metalloproteases [25], suggesting that metalloproteases may also be involved in the cellular activity of other bacterial toxins. However, the possibility remains that multiple proteases, possibly of different classes, are involved in this S1 processing event.

We also determined the inhibitory activity on S1 processing by CHO cells of two inhibitors of cellular trafficking and secretion, bafilomycin A_1_, which inhibits vacuolar proton ATPase and therefore prevents endosome acidification [26], and BFA, which disrupts the Golgi apparatus [10]. Bafilomycin A_1 _had a significant inhibitory effect on S1 processing, with 51% of cell-associated S1 in the unprocessed form (Fig. 2B), consistent with the hypothesis that S1 processing occurs in the endosomal compartment of CHO cells and demonstrating a role for endosome acidification in this processing event. However, BFA had no inhibitory effect on S1 processing (Fig. 2B), consistent with previously reported results [14] and with the hypothesis that S1 processing occurs prior to the Golgi apparatus in the putative retrograde trafficking pathway.

### Effects of S1 processing inhibitors on cellular activity of PT

Proteolytic processing of bacterial toxins is a common theme in their activation within mammalian cells [16], but whether cellular processing of S1 plays a role in the activity of PT had not been previously addressed. As a preliminary investigation of this question, we sought to determine whether the inhibitors of S1 processing by CHO cells had any effect on the ability of PT to ADP-ribosylate target G proteins in CHO cells. CHO cells were preincubated with inhibitors before addition of PT (1 nM) as before. Controls were cells to which either PT or PT* (which has no ADP-ribosylation activity) was added in the absence of inhibitor. Cells were recovered after 3 h, and lysates were prepared and tested in the ADP-ribosylation assay. In this assay, active PT within cells will ADP-ribosylate available G proteins, so that when a lysate is prepared from these cells and used in an in vitro ADP-ribosylation assay with PT and ^32^P-labelled NAD, there is no labeling of G proteins in the lysate, since they were already modified by the PT added to the cells. If PT activity within cells is inhibited, then a proportion of the G proteins in the lysate from these cells will be unmodified and therefore labeled in the in vitro reaction with PT. The assay was repeated twice and the result of one experiment is shown in Fig. 2C. The mean percent inhibition for the various inhibitors (in decreasing order of their inhibitory effect) was as follows: pefabloc SC – 95%, BFA – 79%, bafilomycin A_1 _– 49%, EDTA – 44%, phenanthroline – 28%, 3,4-DCI – 9%, pepstatin – 8%. Therefore there was no strong correlation between the extent of inhibition of S1 processing by the inhibitors and their inhibitory activity on ADP-ribosylation of G proteins by PT in CHO cells. The cell trafficking/secretion inhibitors had a significant inhibitory effect (BFA has previously been shown to inhibit cellular activity of PT [7-9], presumably due to its disruption of PT trafficking in cells, despite its lack of inhibition of S1 processing), but the metalloprotease inhibitors had only a mild inhibitory effect. Of the other protease inhibitors, pefabloc SC had the greatest inhibitory effect, as it did on S1 processing, whereas pepstatin had no significant inhibitory effect on ADP-ribosylation (or S1 processing). To rule out an effect of the inhibitors on the enzymatic activity of PT (independent of its cellular activity), we also performed in vitro ADP-ribosylation assays with PT in the presence of the various inhibitors at the concentrations used on the CHO cells. No significant inhibitory effect was seen with any of the inhibitors (data not shown) with the exception of 3,4-DCI, which was also somewhat toxic to the CHO cells but did not significantly inhibit processing of S1. Altogether, these data are inconclusive with regard to the hypothesis that cellular processing of S1 plays a role in the ADP-ribosylation of G proteins in CHO cells by PT.

### Location of processing site on S1

**Figure 3:**
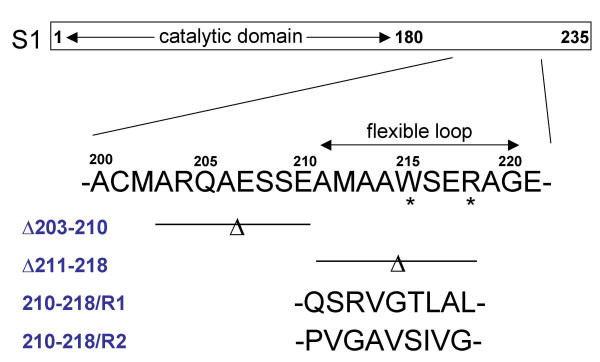
Diagram of the S1 protein and the putative cleavage site. The region of investigation between A200 and E221 is highlighted and details of some of the mutant constructs are shown. The line with a Δ symbol indicates the deleted amino acids.

**Figure 4:**
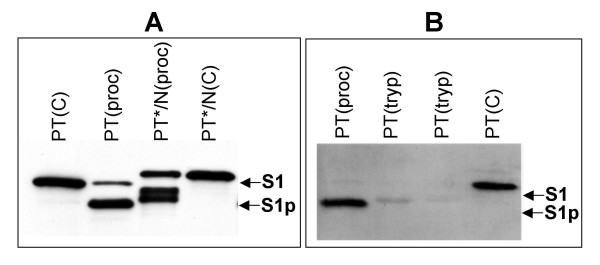
Localization of the processing site on S1. (A) CHO cells were incubated with PT or PT*-CSP/N (PT*/N) for 4 h and analyzed for S1 processing as before. Western blots are shown of the processing samples (proc) or purified protein loading controls (C). (B) Western blot comparing S1p after processing of PT in CHO cells (proc) or trypsin cleavage (tryp) of PT (200 or 100 ng).

We reasoned that if we could identify the precise processing cleavage site on S1, then we would be able to mutagenize this site, obtain a mutant form of S1 that was resistant to cellular processing, and then determine whether processing was required for cellular activity of PT. The size of S1p compared to unprocessed S1 (approximately 22 kDa versus 26 kDa) suggested a cleavage site close to either the N-terminus or the C-terminus of S1, although processing at both termini was also a possibility. Processing at the N-terminus of S1 seems unlikely in view of the fact that the arginine at amino acid 9 (R9) is crucial to enzymatic activity [27]. A protease-sensitive loop that has primary sites for trypsin and chymotrypsin cleavage [15] is located towards the C-terminus of S1 (amino acids 211–220; Fig. 3) [1]. To help define the location on S1 of processing by CHO cells, we compared the cellular processing of a modified form of PT containing an extension of 9 amino acids at the N-terminus of S1 (PT*-CSP/N) to that of native PT. As shown in Fig. 4A, the processed form of S1 in cells incubated with PT*-CSP/N was slightly larger (approximately 1 kDa) than that of cells incubated with PT, leading to the conclusion that processing occurs towards the C-terminus of S1. The presence of 2 processed forms of S1 in cells incubated with PT*-CSP/N presumably reflects some additional processing of the N-terminal extension, but since each of these forms is larger than that of S1p from native PT, the conclusion is the same. Fig. 4B shows that the major trypsin-digested fragment of S1 is slightly larger than S1p from cellular processing of PT, suggesting that the site on S1 of cellular processing is close to the protease-sensitive loop, but is N-terminal to the trypsin cleavage site (at R218). However, we cannot rule out the possibility of intracellular modification of S1 that would alter its migration on SDS-PAGE gels and complicate the interpretation of this experiment.

**Figure 5:**
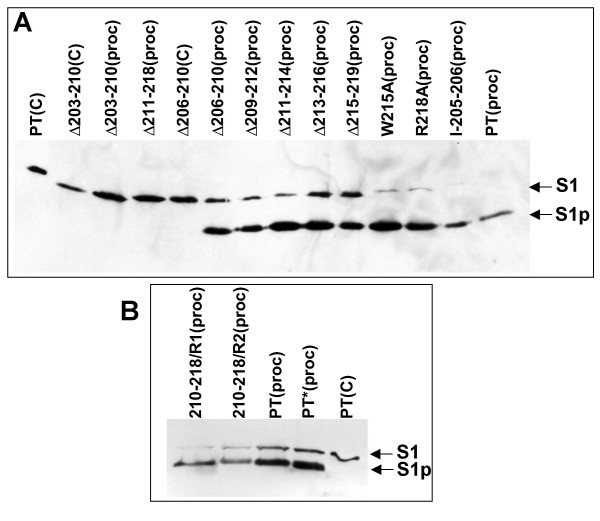
Processing of mutant PT proteins in CHO cells. (A) Western blot showing purified protein (lanes marked [C]) or processing of S1 (lanes marked [proc]) after addition of various mutant PT proteins (20 nM) to CHO cells. (B) Western blot showing processing of S1 (lanes marked [proc]) after addition of the 210–218/R replacement mutant PT proteins or PT and PT* controls to CHO cells.

We performed extensive site-specific mutagenesis in the region between M202 and R218 of S1 in an attempt to obtain a mutant form of PT that was no longer processed in mammalian cells. However, we were unable to obtain such a mutant by this approach – almost all PT mutants were processed normally, including those with changes at the primary cleavage sites for trypsin (R218A) and chymotrypsin (W215A) (Fig. 5A). Certain changes at residue A203 (A203D, A203R) resulted in very low levels (<1%) of PT secretion by *B. pertussis *(data not shown) so that we were unable to determine whether processing was affected. However, other changes at this residue (A203G, A203S, A203V) had no effect on PT secretion, processing or activity (data not shown). In an attempt to locate the processing site, we constructed mutant forms of PT with deletions of various stretches of residues in this region. Each of 2 adjacent deletions of 8 residues (Δ203–210, Δ211–218; Fig. 3) resulted in the complete loss of S1 processing in CHO cells (as far as could be determined by western blotting – Fig. 5A), and the same was true of the overlapping 8 residue deletion Δ207–214 (data not shown), strongly suggesting that the processing site is located in this region. However, smaller overlapping deletions of 4 or 5 residues in this region did not disrupt S1 processing (Fig. 5 and data not shown), indicating that no particular sequence in this region served as a specific cleavage site for the processing event. In further support of this idea, 2 PT constructs in which residues 210–218 of S1 were replaced by random amino acid sequence (210–218/R1 and 210–218/R2) were both processed normally (Fig. 5B). Equivalent replacement of residues 203–210 abrogated assembly and secretion of PT by *B. pertussis*, so we could not make the same assessment for this region. However, two lines of evidence suggested that the processing site may be located in the region of residue A203. First, a PT construct (I-205–206) with an insertion of 8 amino acids between residues Q205 and A206 in S1 was processed to the same size S1p as wild type PT (Fig. 5A), indicating that the processing site was upstream of the insertion (although it may have been within the insertion). Second, mass spectrometry analysis of the processed and unprocessed forms of S1 in lysates from PT-treated CHO cells identified a difference in mass between S1 and S1p (3577.4) most closely corresponding to the theoretical mass of a peptide from R204 to the C-terminus of S1 (3603.9) (data not shown). However, as mentioned above, substitutions at A203 either did not affect processing of S1 or greatly reduced secretion of PT, and substitutions at M202 and R204 also did not affect S1 processing (data not shown), so we were unable to verify this putative cleavage site by mutagenesis. It is also possible that the major processing site is further downstream (within the 211–220 loop region for example) with subsequent additional cleavage occurring during cell lysis, resulting in the apparent size of S1p.

### Effect of processing site mutations on PT activity

**Figure 6:**
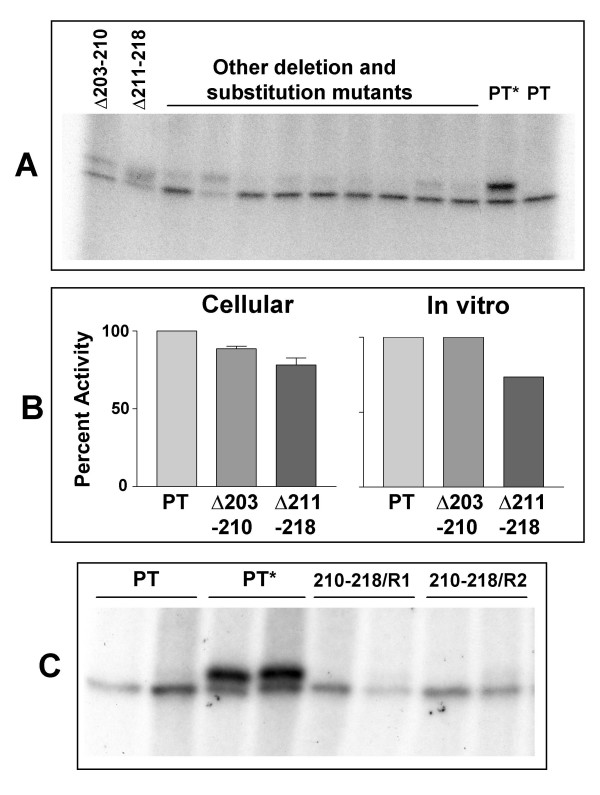
ADP-ribosylation assay of mutant PT constructs. (A) Autoradiogram of cellular ADP-ribosylation assay after treatment of CHO cells with the indicated PT protein. The upper radiolabeled band (approximately 41 kDa) corresponds presumably with G_i_α2 and G_i_α3, while the lower band is PT-independent endogenously labeled band often seen in this assay with CHO cell lysates [9]. (B) Graphed data showing the mean (n = 3) cellular activity of the 2 deletion constructs that are not processed (activity of wild type PT was set at 100%) and the in vitro ADP-ribosylation activity of the same proteins. (C) Autoradiogram of cellular ADP-ribosylation assay after treatment of CHO cells with the indicated PT protein, demonstrating the retention of activity of the replacement mutant PT constructs.

We determined the effect of several of these deletion and replacement mutations on PT activity using the ADP-ribosylation assay. Cells were incubated with the purified PT construct (2 nM) for 3 h to determine cellular activity. As shown in Fig. 6A, all mutant constructs retained significant activity, relative to the inactive mutant PT*. The two deletion mutants that were not processed in cells were analyzed further and were found to possess 88.5% (Δ203–210) and 78.1% (Δ211–218) of wild type PT cellular activity (Fig. 6B). The in vitro activity of these constructs was also assayed, using a fusion of GST with the C-terminal 20 amino acids of human Giα3 (GST-αC20) as the substrate, with the finding that the Δ203–210 mutant possessed 100% of wild type PT activity while the Δ211–218 mutant had a 26.5% reduction in activity (Fig. 6B). We also found that the 210–218 replacement mutants retained full cellular activity (Fig. 6C). Together these data demonstrate that S1 processing is not essential for PT cellular activity, since the mutants in which processing was apparently abolished retained significant activity. However, this conclusion must remain tentative in the absence of a single substitution mutant that is unprocessed, which would be the best reagent to answer this question. A large deletion of eight amino acid residues may affect other PT-associated properties or may mask the effects of the loss of processing. The stability of these mutant forms of PT may have been altered, though apparently not significantly reduced from the processing assay data (Fig. 5). It is also possible that the number of PT molecules necessary for full activity is low enough to be undetectable in the processing western blot assay. In previous studies, limited trypsin cleavage of PT increased its activation in vitro [15], indicating that S1 processing may play a role in activity, and domains on S1 for interaction with target proteins and enzymatic activity were found to lie within the N-terminal 204 amino acids [28], so the processed form of S1 should retain these functions.

We also tested several strains expressing mutant PT constructs in our mouse infection model of *B. pertussis *virulence [29], and none of these strains was significantly defective in colonization compared to the parental wild type strain (data not shown). Although these data suggest that S1 processing is not essential for the virulence of *B. pertussis*, it is possible that S1 could be processed in vivo by alternative proteases absent from CHO cells.

### Processing in stable CHO cell transfectants expressing S1

**Figure 7:**
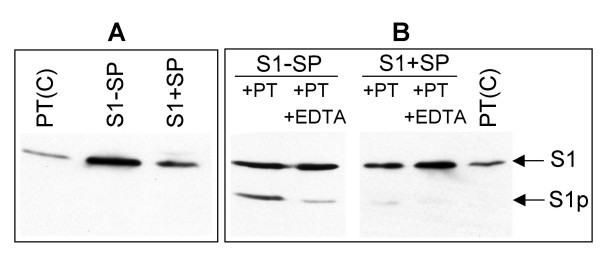
S1 processing in CHO cell transfectants expressing S1. (A) Western blot showing lack of processing of endogenous S1 in transfectants expressing S1 in the cytosol (S1-SP) or in the ER (S1+SP). (B) Western blot showing EDTA-inhibitable processing of S1 after exogenous addition of purified PT (20 nM) to these transfectants.

Previously we constructed stable CHO cell transfectants expressing S1 either with a signal peptide (for ER localization) or without one (for cytoplasmic localization), and showed that S1 fully ADP-ribosylated target G proteins in each transfectant [12]. In that study we did not observe significant processing of S1 in whole cell lysates of these transfectants, but we re-examined this issue using Triton X-100 lysis of transfectants and western blotting of the insoluble pellet material. As seen in Fig. 7A, there was no detectable processing of S1 to S1p in either transfectant. Exogenous addition of PT to these cells resulted in EDTA-inhibitable processing of S1 to S1p (Fig. 7B), demonstrating that S1 expression and activity in the transfectants did not prevent S1 processing of exogenously added PT, though the level of processing in the S1+SP transfectant was apparently quite low.

The data from these transfectants do not rule out the possibility that S1 processing may contribute to the trafficking of PT to the ER. However, the data are consistent with the idea that S1 processing is not essential for its activity in mammalian cells, and therefore that translocation of the full-length S1 across the ER membrane occurs. This would distinguish PT from several other bacterial toxins of similar subunit structure, such as cholera toxin, heat-labile toxin and shiga toxin, for which processing of the A subunit is apparently important for cellular activity [16]. The difference may be in the association of the A subunit with the B oligomer. The other toxin A subunits have a relatively long helix that protrudes through a central pore in the B oligomer [30], and cleavage of the A subunit is required to release the enzymatic domain from this complex. S1 has a relatively short helix associated with the B oligomer [1], and therefore cleavage may be unnecessary for its release from the holotoxin complex in the ER, or whichever compartment translocation occurs from.

## Conclusions

In this study we have further characterized the cellular processing of the S1 subunit after PT interacts with mammalian cells. Our major conclusion is that this processing event is not essential for PT activity in mammalian cells, based on several lines of evidence: (1) protease inhibitor studies showed a general lack of correlation between S1 processing and PT cellular activity; (2) mutant forms of PT in which S1 processing was apparently abolished retained significant (>75% of wild type) cellular activity; and (3) no S1 processing was apparent in transfected cells expressing active S1. Although we have not definitively ruled out a contribution of S1 processing to the cellular activity of PT due to the imperfect nature of our unprocessed mutants, it is possible that the processing event is completely unrelated to PT cytotoxicity and instead is an irrelevant activity occurring, possibly in lysosomes, on the large majority of the intracellular pool of PT molecules that do not enter the putative retrograde transport pathway to the ER and then on to the cytosolic target proteins.

## Methods

### Bacterial strains and growth conditions

The *B. pertussis *strain used in this study was a streptomycin- and nalidixic acid-resistant derivative of W28 (Wellcome). The PT9K/129G (PT*) derivative of this strain was constructed as previously described [27]. The other PT mutant derivatives used in this study were constructed as described below. *B. pertussis *was grown on Bordet-Gengou agar (Difco) plates containing 15% defibrinated sheep blood and the following antibiotics at the indicated concentrations where necessary: streptomycin 400 μg ml^-1^, nalidixic acid 20 μg ml^-1^, gentamicin 10 μg ml^-1^; or in Stainer-Scholte liquid medium [31] containing heptakis-dimethylcyclodextrin (Sigma). *Escherichia coli *strains used were DH10B [32] for standard cloning experiments and S17.1 [33] for conjugation with *B. pertussis*, and these were grown on LB agar plates containing 10 μg ml^-1^gentamicin where necessary or in LB broth containing 100 μg ml^-1^ampicillin.

### Plasmid and strain construction

Plasmid pJ-PT was obtained by subcloning a 2.3 kb *Eco*RI-*Bsr*GI fragment (containing the PT S1 and S2 genes) into *Eco*RI/*Acc*65I-digested allelic exchange vector plasmid pJHC1 [34]. Mutations were engineered into the S1 sequence of this plasmid, which was confirmed by DNA sequencing, transformed into *E. coli *S17.1 and introduced into the *B. pertussis *chromosome by conjugation and allelic exchange as described previously [35]. Deletion, insertion and substitution mutations were constructed by overlap extension PCR [36], and the 210–218/R1 and R2 replacement mutations were constructed by using a degenerate oligonucleotide (CARBON 1097; 5'-GATAAGAGCTCCVNNVNNVNNVNNVNNVNNVNNVNNVNNGCCGGCGAGGCCTCGCC-3' where V = G, A, or C and N = any nucleotide) which was allowed to anneal, extended with DNA polymerase Klenow fragment, digested with SacI and BglI and inserted into SacI- and BglI-digested pJ-PT. The GST-αC20 construct was obtained by inserting annealed complementary oligonucleotides (encoding the C-terminal 20 amino acids of human Giα3) into the plasmid pGEX-2T (Pharmacia) to derive pGEX-αC20. The PT*-CSP/N construct was obtained as previously described [11].

### Western blotting

Samples were run on 12% SDS-PAGE gels and transferred to nitrocellulose filters. To detect S1, blocked filters were incubated with S1-specific monoclonal antibody X2X5 or 1C7, followed by peroxidase conjugated anti-mouse IgG secondary antibody (Amersham). Blots were developed using ECL Plus (Amersham) and exposed to X-ray film.

### Protein purification

PT and mutant derivatives were prepared from *B. pertussis *culture supernatants by the fetuin affinity method of Kimura et al. [37], resuspended in PBS and stored at -80°C until use. The protein concentration was determined by BCA assay (Pierce). GST-αC20 protein was purified from a culture of *E. coli *DH10B containing the plasmid pGEX-αC20. For induction of the fusion protein, the strain was grown in LB to A600 1.0 and then IPTG was added to a concentration of 0.5 mM. 2 h after IPTG addition, cells were centrifuged, lysed in BPER lysis reagent (Pierce), cleared by centrifugation, and passed through a GSTrap column (Amersham-Pharmacia). Fusion protein was eluted in reduced glutathione buffer, dialyzed against PBS and analyzed by SDS-PAGE and BCA assay (Pierce) to determine protein concentration.

### Cell lysis and fractionation

CHO cells were grown in 6-well plates to near confluency and then PT was added and incubated for the indicated times at 37°C. Cells were then collected by trypsinization, washed in PBS, resuspended in 50–100 μl of either NET/Triton lysis buffer (150 mM NaCl, 5 mM EDTA, 50 mM Tris, pH 7.4, 0.01% NaN_3_, and 0.5% Triton X-100) or RIPA buffer (10 mM Tris, pH 7.4, 0.1% SDS, 1% sodium deoxycholate, 1% NP-40, 150 mM NaCl) and incubated 30 min on ice. The lysate was then centrifuged 15 min at 13,000 rpm at 4°C in a microfuge, the supernatant was removed to a fresh tube and the pellet was resuspended in sample buffer. Samples were boiled 5 min and loaded onto an SDS-PAGE gel.

### Inhibitors

Inhibitors were added to CHO cells 30 min prior to the addition of PT. For protease inhibitors we followed the suggestions of Barrett [24] to determine the catalytic type of protease involved. Protease inhibitors (BMB/Roche) and concentrations used were: aprotinin (0.15 μM), 3,4-dichloroisocoumarin (3,4-DCI, 1 mM) and 4-(2-aminoethyl)-benzenesulfonyl-fluoride, hydrochloride (pefabloc SC, 1 mM) for inhibition of serine proteases; trans-epoxysuccinyl-L-leucylamido(4-guanidino)butane (E-64, 1 mM) and leupeptin (1 μM) for inhibition of cysteine proteases; pepstatin (1 μM) for inhibition of aspartic proteases; and EDTA (0.5–1 mM) and 1,10-phenanthroline (0.5–1 mM) for inhibition of metalloproteases. Other inhibitors used were BFA (Sigma, 5 μg/ml) and bafilomycin A_1 _(ICN, 0.5 μM). After SDS-PAGE and western blotting of samples, band intensities were measured by densitometry and used to calculate the extent of inhibition of S1 processing.

### Trypsin digestion of PT

PT (100 or 200 ng) was digested with trypsin (Sigma) in a volume of 20 μl at room temperature for 1 h in 50 mM Tris, pH 8, and 2 mM CaCl_2_. Trypsin was present at 70 μg/ml. Sample buffer was added and the samples were then boiled 5 min before loading onto an SDS-PAGE gel.

### Western blotting

Samples were run on 12% SDS-PAGE gels and transferred to nitrocellulose filters. To detect S1, blocked filters were incubated with S1-specific monoclonal antibody X2X5 (3) (a generous gift from Drusilla Burns) or 3CX4, followed by peroxidase conjugated anti-mouse IgG secondary antibody (Amersham). Blots were developed using ECL Plus (Amersham) and exposed to X-ray film.

### ADP-ribosyltransferase assays

To determine the cellular activity of PT samples, PT (0.5–2 nM) was added to near confluent CHO cells in 12-well culture plates, and after 3 h at 37°C cells were recovered from plates, washed in PBS and lysed in NET/Triton lysis buffer 30 min on ice. The lysate was then centrifuged 15 min at 13,000 rpm at 4°C and the supernatant was stored at -20°C until the assay. The ADP-ribosylation assay contained, in 25 μl, 0.1 M Tris, pH 7.5, 20 mM dithiothreitol (DTT), 0.5 mM ATP, 1 μM ^32^P-NAD (specific activity 30 Ci/mmol; NEN), 10 ng PT, and an aliquot of the lysate sample. For assessment of in vitro enzymatic activity of PT samples by ADP-ribosylation assay, reactions contained, in 25 μl, 0.1 M Tris, pH 7.5, 20 mM DTT, 0.5 mM ATP, 1 μM ^32^P-NAD (specific activity 30 Ci/mmol; NEN), 10–50 ng PT, and 0.5 μg GST-αC20 protein as substrate. The mixture was incubated 90 min at room temperature, sample buffer was added, and the sample was boiled 5 min and loaded onto 15% SDS-PAGE gels. After electrophoresis, gels were fixed, dried and exposed to X-ray film. Band intensities were measured by densitometry and used to calculate the extent of ADP-ribosylation of target proteins.

### Mass spectrometry analysis

CHO cells were incubated overnight with PT and then lysates were made using Triton X-100 buffer at room temperature (to maximize the proportion of S1 in the soluble fraction). Purified 1C7 monoclonal antibody was allowed to bind to a PS10 chip (Ciphergen Biosystems) for 2 h at room temperature, which was then washed with PBS. The CHO cell lysates (soluble fraction) were diluted 1:1 with PBS and 100 μl of each lysate was added to a spot on the chip and incubated at 5°C for 4 days. The chip was washed 3 times with PBS containing 0.1% Triton X-100, 3 times with PBS, and twice with 5 mM HEPES (pH 7.0). After the chip was air-dried, matrix (Sinapinic acid) was added to each spot and allowed to dry. Matrix-assisted laser desorption/ionization-time of flight (MALDI-TOF) mass spectrometry analysis of these samples was performed in a model PBS-II machine (Ciphergen).

### Mouse infection

Six-week-old female BALB/c mice (Harlan) were used for infection experiments. Bacterial inocula were prepared and intranasal inoculation of mice was performed as previously described [29]. Seven days after inoculation, mice were sacrificed by carbon dioxide inhalation and the respiratory tract (trachea + lungs) was removed, homogenized in 2 ml PBS, and dilutions were plated on BG-blood agar plates containing streptomycin to determine the number of colony forming units (CFU) per respiratory tract.

## Authors' contributions

NHC conceived of the study, performed some experiments, supervised personnel and drafted the manuscript. RMM performed most of the plasmid and strain constructions, protein purification, processing assays and cell culture work. GVA performed the mouse infection experiments, some plasmid and strain constructions, protein purification, processing assays and ADP-ribosylation assays. RDP performed some of the plasmid and strain constructions and processing assays. ZEVW performed the GST fusion constructions and purification, some ADP-ribosylation assays, cell culture work and contributed to supervision of the other personnel.
